# Precise Target Geo-Location of Long-Range Oblique Reconnaissance System for UAVs

**DOI:** 10.3390/s22051903

**Published:** 2022-02-28

**Authors:** Xuefei Zhang, Guoqin Yuan, Hongwen Zhang, Chuan Qiao, Zhiming Liu, Yalin Ding, Chongyang Liu

**Affiliations:** 1 Changchun Institute of Optics, Fine Mechanics and Physics, Chinese Academy of Sciences, Changchun 130033, China; zhangxuefei@ciomp.ac.cn (X.Z.); yuanguoqin@ciomp.ac.cn (G.Y.); zhanghongwen@ciomp.ac.cn (H.Z.); dingyalin@ciomp.ac.cn (Y.D.); liuchongyang@ciomp.ac.cn (C.L.); 2 Key Laboratory of Airborne Optical Imaging and Measurement, Chinese Academy of Sciences, Changchun 130033, China; 3 Beijing Institute of Control Engineering, Beijing 100190, China; qiaochuan@163.com

**Keywords:** unmanned aerial vehicles, long-range oblique reconnaissance system, target geo-location, cubature Kalman filtering

## Abstract

High-precision, real-time, and long-range target geo-location is crucial to UAV reconnaissance and target strikes. Traditional geo-location methods are highly dependent on the accuracies of GPS/INS and the target elevation, which restricts the target geo-location accuracy for LRORS. Moreover, due to the limitations of laser range and the common, real time methods of improving the accuracy, such as laser range finders, DEM and geographic reference data are inappropriate for long-range UAVs. To address the above problems, a set of work patterns and a novel geo-location method are proposed in this paper. The proposed method is not restricted by conditions such as the accuracy of GPS/INS, target elevation, and range finding instrumentation. Specifically, three steps are given, to perform as follows: First, calculate the rough geo-location of the target using the traditional method. Then, according to the rough geo-location, reimage the target. Due to errors in GPS/INS and target elevation, there will be a re-projection error between the actual points of the target and the calculated projection ones. Third, a weighted filtering algorithm is proposed to obtain the optimized target geo-location by processing the reprojection error. Repeat the above process until the target geo-location estimation converges on the true value. The geo-location accuracy is improved by the work pattern and the optimization algorithm. The proposed method was verified by simulation and a flight experiment. The results showed that the proposed method can improve the geo-location accuracy by 38.8 times and 22.5 times compared with traditional methods and DEM methods, respectively. The results indicate that our method is efficient and robust, and can achieve high-precision target geo-location, with an easy implementation.

## 1. Introduction

Unmanned aerial vehicles (UAVs) have attracted widespread attention, with the advantages of good timeliness and flexibility. In the military, UAVs have been demonstrated to be effective mobile platforms, which can satisfy the spatial and temporal resolution requirements for carrying onboard sensors [1,2,3,4]. In civil applications, numerous UAV platforms have been applied to disaster monitoring, rescue, mapping, surveying, and environment scouting [5,6,7,8]. In order to enhance reconnaissance security during high-risk missions and achieve a wide area search, long-range oblique reconnaissance systems (LRORS) and high precision, real-time target geo-location have become prevalent [9,10,11,12,13,14].

The geo-location method is essential to achieve high-precision geo-location. To obtain the geo-location of the ground target, target geo-location algorithms have been widely studied by many scholars. D. B. Barber [15] proposed a localization method based on the flat earth model for a ground target when imaged from a fixed-wing miniature air vehicle (MAV). This method has a good localization effect for low-attitude and short-range targets. The experiment results showed that it could improve the localization of the target to within 3 m when the MAV flew at 100–200 m. However, this method is not applicable for LRORS, since the influence of the earth curvature was not considered.

In view of the above problem, J. Stich [16] proposed a target geo-location method based on the World Geodetic System 1984 (WGS84) ellipsoidal earth model. This method is also the traditional method used by LRORS at present. This algorithm enables autonomous imaging of defined ground areas at arbitrary standoff distances, without range finding instrumentation, and effectively reduces the influence of earth curvature on the target geo-location accuracy. The method calculates the geo-location based on the external orientation elements measured by GPS/INS and the elevation of the target. On the one hand, the geo-location accuracy depends on similar ground elevations under the platform and at the target area. For mountainous regions, the allowable stand-off distance will be constrained by the variation in terrain elevation. The impact of the terrain elevation on geo-location accuracy was analyzed in [17]. The results demonstrated that with the increase of off-nadir looking angle and imaging distance, the influence of target elevation on the geo-location accuracy increases. On the other hand, the measurement accuracy of the GPS/INS equipped by most UAVs is not sufficient to achieve high precision geo-location. Since the target elevation is usually unknown in an actual project, and usually the GPS/INS is not sufficient, application of the traditional method is limited.

In order to improve the geo-location precision when the target elevation is unknown, the laser range finder (LRF) method [18,19] is proposed. By measuring the distance between the target and the UAVs, the LRF method resolves the influence of target elevation on geo-location accuracy. Flight-test results show that the target geo-location accuracy is less than 8 m when the distance between the target and the UAVs is 10 km [18]. However, the LRF method is not suitable for target geo-location of LRORS, due to the limitation of laser range [19].

The digital elevation model (DEM) method [20,21] can solve the distance limitation of the LRF method. A method based on DEM was described in [20]. The simulation and the flight-test results demonstrated that when the off-nadir looking angle is 80 degrees and the target ground elevation is 100 m, the geo-location accuracy can be improved, from the 600 m of the traditional method, to 180 m with the DEM method. However, the DEM method has some problems in practical applications for LRORS. First, the DEM method is restricted by the accuracy and continuity of DEM data. Second, the DEM method cannot be used in artificial buildings, because usually the DEM data do not contain the height information of buildings. Third, the DEM method needs pre-obtained data and has a high computational cost. The DEM method is inappropriate for real-time target geo-location.

In order to solve the problem that DEM data does not include the height information of a building, a geo-location algorithm for building targets, based on the image method, was proposed in [22]. A convolutional neural network was used to automatically detect the location of buildings, and the imaging angle was used to estimate the height of a building. The results demonstrated that the image method can improve the positioning accuracy of building targets by approximately 20–50% compared with the traditional geo-location method. Unfortunately, this method is also inappropriate for real-time target geo-location because of the requirements of the complexity of image processing.

Methods based on geographic reference data [23,24,25,26] and cooperative localization between UAVs [27,28,29,30] have been proposed to improve the accuracy of the target geo-location. These methods are also inappropriate for real-time target geo-location because of the need for pre-obtained geo-referenced images and high flight cost.

Focusing on these above mentioned problems, we propose a novel geo-location method for long-range UAVs, which uses multiple observations on the same target by a single UAV to improve the geo-location accuracy. The main contributions of this paper are given as follows:

1. Based on a comparative analysis of the present methods affecting geo-location accuracy, a set of work patterns and a novel geo-location method are proposed in this paper to address these problems. There is an iterative process in the proposed method, and the geo-location accuracy is improved greatly by repeatedly imaging the same stationary target point. In brief, the procedure can be summarized by the following: Step 1, calculate the rough geo-location of the target using the traditional method. Step 2, based the rough geo-location of the target, adjust the position of the gimbal and reimage the target. Step 3, process the reprojection errors and obtain optimized target geo-location. Repeat the above process; after several iterations, the estimated geo-location converges on the true value.
2. Compared with the traditional method, the proposed method does not rely on the accuracy of GPS/INS and target elevation, which are regarded as the key error sources in the traditional method. Compared with the laser range finder method, the proposed method is not limited by laser ranging distance. Compared with the DEM method and image method, high-precision real-time geo-location can be realized without DEM or geographic reference data. Compared with cooperative localization between UAVs, the proposed method can achieve high precision without multiple UAVs.
3. The proposed method can achieve high precision, without high-precision GPS/INS, multiple UAVs, and geographic reference data, such as a standard map, DEM, and so on. The proposed method has strong timeliness and a more extensive application value in practical engineering.

The remainder of the paper is structured as follows: Section 2 introduces the traditional geo-location model, based on the WGS-84 ellipsoidal earth model. Section 3 elucidates the detailed implementation of the proposed work pattern and algorithm. Finally, experimental validation is presented in Section 4, and the discussions associated with the experimental results and analysis are organized in Section 5, while conclusions are drawn in Section 6.

## 2. Geo-Location Method Based on WGS-84 Ellipsoidal Earth Model

This section first introduces the traditional geo-location method based on the WGS-84 ellipsoidal earth model. Then, the sources of influence on the geo-location accuracy of the traditional geo-location method are analyzed.

### 2.1. Geo-Location Model using the Traditional Method

Four basic coordinate systems are used in the traditional geo-location method, including the earth-centered earth-fixed (ECEF) coordinate system, the north-east-down (NED) coordinate system, the UAV platform (*P*) coordinate system, and the sensor (*S*) coordinate system, respectively.

In the following discussion, the coordination of point *D* in the *A* coordinate system is denoted as $D_{A}={\left[\begin{array}{ccc} x_{D}^{A} & y_{D}^{A} & z_{D}^{A} \end{array}\right]}^{T}$, coordination transforms are denoted by $C_{A}^{B}$, where *C* is the matrix transformation from *A* coordination system to *B* coordination system, $C_{B}^{A}={\left(C_{A}^{B}\right)}^{-1}$ is the matrix transformation from *B* coordination system to *A* coordination system. The transformation matrices for each of the coordinate systems are introduced in Appendix A.

**Figure 1:**
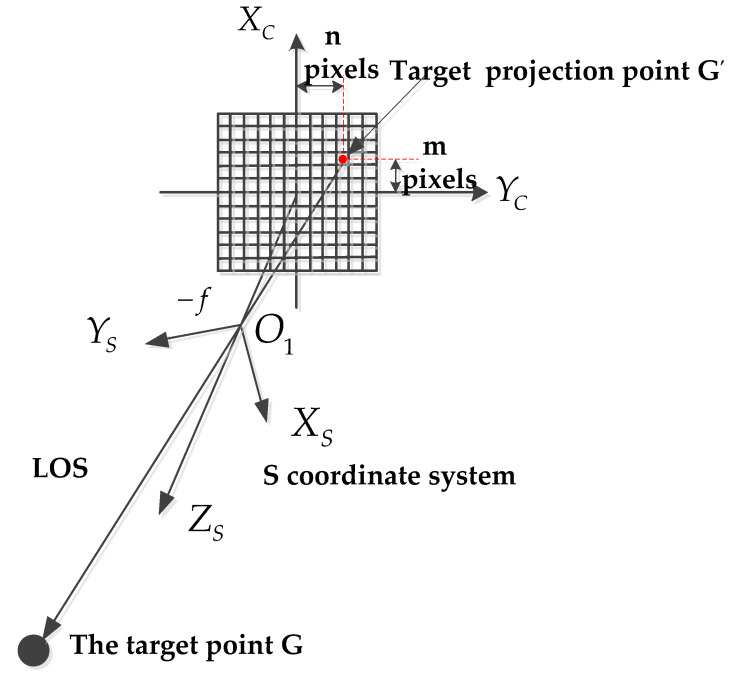
The projection of the target point *G* on a frame CCD.

The target point *G* is projected on a frame CCD, as shown in Figure 1. A frame CCD is made of a 2D array of sensor detectors, and one exposure captures the entire scene. The deviation of the projection point from the CCD center is m pixels and n pixels in the *X_C_* and *Y_C_* direction.

The projection point *G*′ in *S* coordinate system can be expressed as

(1) $$G_{S}^{\prime }={\left[\begin{array}{ccc} x_{G^{\prime }}^{S} & y_{G^{\prime }}^{S} & z_{G^{\prime }}^{S} \end{array}\right]}^{T}={\left[\begin{array}{ccc} m\times a & n\times a & -f \end{array}\right]}^{T},$$

As shown in Equation (1), a is the pixel size of the CCD, and f is the focal length of LRORS. The target projection point *G*′ and the origin of the *S* coordinate system *O*_1_ in the ECEF coordinate system can be expressed as *G*′*_E_* and *O*_1*E*_.

(2) $$\begin{array}{l} \left[\begin{array}{c} G_{E}^{\prime }\\ 1 \end{array}\right]=\left[\begin{array}{c} \begin{array}{c} x_{G^{\prime }}^{E}\\ y_{G^{\prime }}^{E}\\ z_{G^{\prime }}^{E} \end{array}\\ 1 \end{array}\right]=C_{NED}^{ECEF}\times C_{P}^{NED}\times C_{S}^{P}\times \left[\begin{array}{c} G_{S}^{\prime }\\ 1 \end{array}\right]\\ \left[\begin{array}{c} O_{1E}\\ 1 \end{array}\right]=\left[\begin{array}{c} \begin{array}{c} x_{O_{1}}^{E}\\ y_{O_{1}}^{E}\\ z_{O_{1}}^{E} \end{array}\\ 1 \end{array}\right]=C_{NED}^{ECEF}\times \left[\begin{array}{c} \begin{array}{c} 0\\ 0\\ 0 \end{array}\\ 1 \end{array}\right] \end{array},$$

The target point *G* in the ECEF coordinate system can be expressed as $G_{E}={\left[\begin{array}{ccc} x_{G}^{E} & y_{G}^{E} & z_{G}^{E} \end{array}\right]}^{T}$ and the target to sensor vector can be expressed as $\vec{G_{E}O_{1E}}$, the geodetic height of the target point *G* is defined as *h_G_*. For an ideal LRORS, the target point *G*, the origin of the *S* coordinate system *O*_1_, and the projection point *G*′ are collinear. *G_E_* should meet the condition in Equation (3) [11].

(3) $$\{\begin{array}{l} \frac{x_{G}^{E}-x_{O_{1}}^{E}}{x_{G^{\prime }}^{E}-x_{O_{1}}^{E}}=\frac{y_{G}^{E}-y_{O_{1}}^{E}}{y_{G^{\prime }}^{E}-y_{O_{1}}^{E}}=\frac{z_{G}^{E}-z_{O_{1}}^{E}}{z_{G^{\prime }}^{E}-z_{O_{1}}^{E}}\\ \frac{{\left(x_{G}^{E}\right)}^{2}}{{\left(R_{E}+h_{G}\right)}^{2}}+\frac{{\left(y_{G}^{E}\right)}^{2}}{{\left(R_{E}+h_{G}\right)}^{2}}+\frac{{\left(z_{G}^{E}\right)}^{2}}{{\left(R_{P}+h_{G}\right)}^{2}}=1 \end{array},$$

We can obtain the condition of the target $G_{E}=\left[\begin{array}{ccc} x_{G}^{E} & y_{G}^{E} & z_{G}^{E} \end{array}\right]$ in the ECEF coordinate system from Equation (3). Then, the geo-location ${\left[\begin{array}{ccc} \lambda_{G} & \varphi_{G} & h_{G} \end{array}\right]}^{T}$ of target *G* can be solved according to Equations (A2) and (A3) in Appendix A.

### 2.2. The Sources of Influence in the Traditional Method on Geo-Location Accuracy

**Figure 2:**
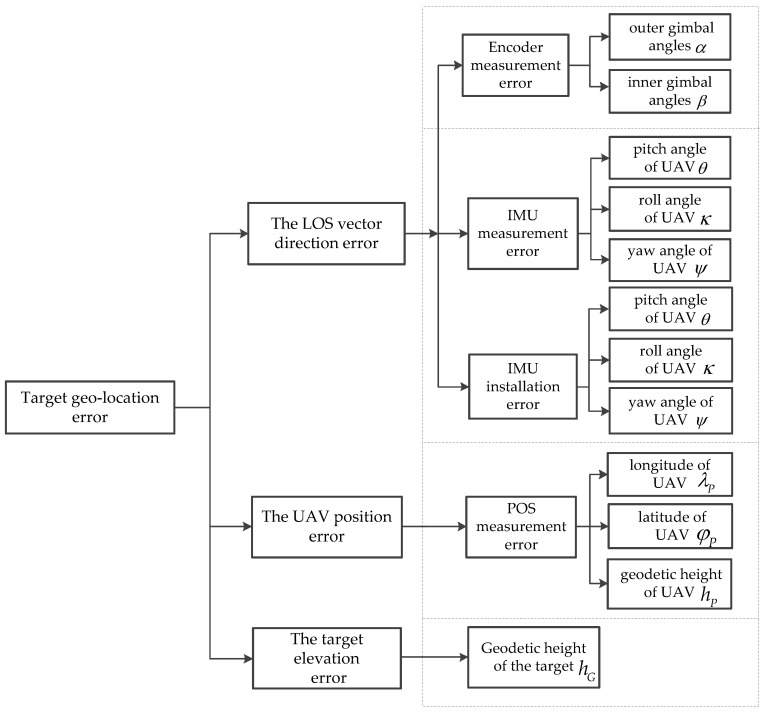
The error sources affecting the geo-location accuracy.

The target geo-location accuracy is affected by the target elevation error and the measurement variances. The target elevation is usually unknown in an actual project. The measurement variances are largely dependent on the measurement precision of the LOS vector direction and the UAV position. LOS vector direction consists of the UAV attitude and the gimbal angles, which are measured by INS and encoders, respectively. The position information of the UAV is measured by GPS. The error sources affecting the geo-location accuracy are shown in Figure 2.

In order to intuitively illustrate the influence of the measurement variances and target elevation error on the geo-location accuracy of LRORS, we conducted comparative analysis on the geo-location accuracy when the off-nadir looking angle was 0°, 35°, and 75°, respectively. The measurement variances in the geo-location are shown in Table 1.

**Figure 3:**
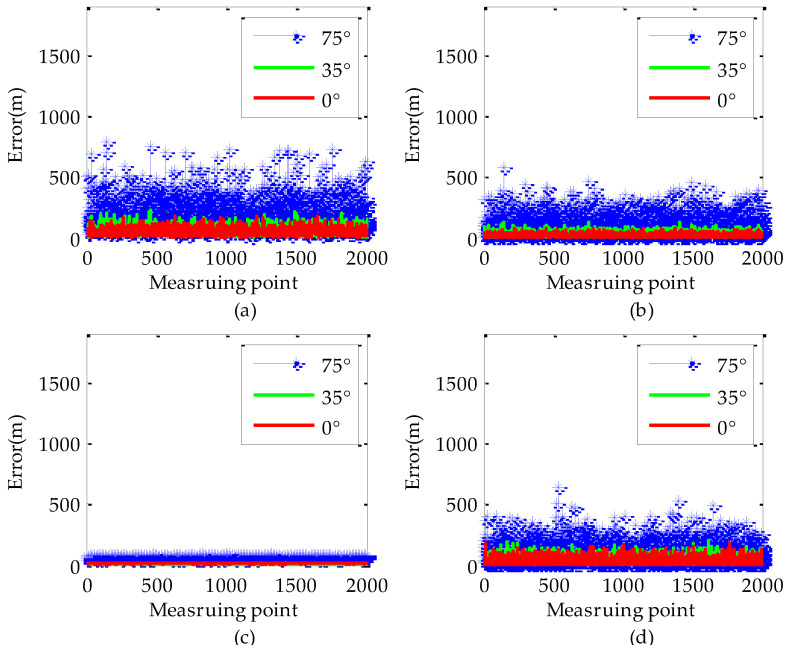
Geo-location error of the target by the traditional method: (**a**) Geo-location error of the target; (**b**) UAV position error in the geo-location; (**c**) LOS vector direction error in the geo-location; (**d**) Target elevation error in the geo-location.

**Figure 4:**
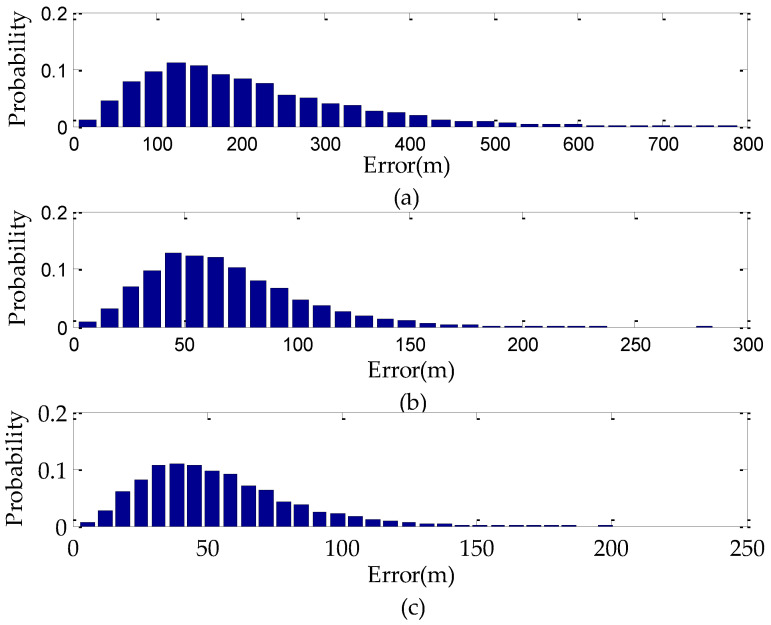
Probability of geo-location error by the traditional method: (**a**) The off-nadir looking angle is 75°; (**b**) The off-nadir looking angle is 35°; (**c**) The off-nadir looking angle is 0°.

Assuming the target *G* is located at the position (43.300000° N, 84.200000° E, 1551.00 m), the target elevation error is 50 m and the UAV flies at a geodetic height of 10000 m. The Monte-Carlo method is used to analyze the target geo-location error. The results are shown in Figure 3 and Figure 4. It can be seen that the geo-location accuracy decreases significantly with the increase of the off-nadir looking angles at the same measurement variances. The maximum value of the geo-location error reaches 800 m when the off-nadir looking angle is 75°, while the maximum value of the geo-location errors are only 300 m and 200 m when the off-nadir looking angles are 35° and 0°, respectively. Among the various measurement variances, the target elevation error has the greatest impact on the geo-location accuracy. When the target elevation error is 50 m, the maximum value of the geo-location errors caused by target elevation error are 130 m, 150 m, and 500 ms at 0°, 35°, and 75° of the off-nadir looking angles, respectively.

## 3. The Proposed Geo-Location Method

In this section, we first introduce the work pattern of the proposed method, then we use the proposed algorithm to estimate the target geo-location.

### 3.1. The Work Pattern of LRORS

**Figure 5:**
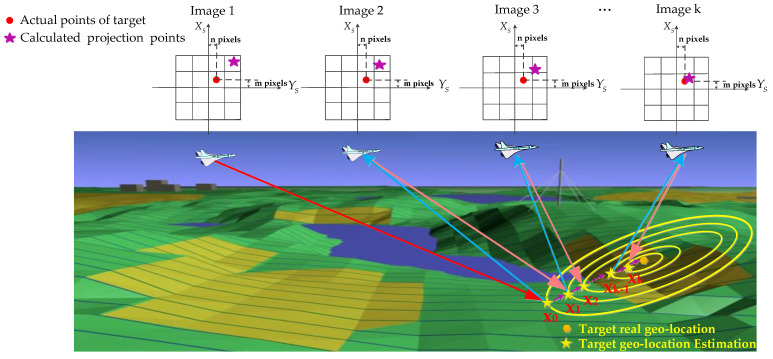
The proposed work pattern.

The work pattern proposed in this paper is shown in Figure 5. First, the initial geo-location of the target is calculated using the traditional method. Affected by the accuracy of GPS/INS and the target elevation, the initial geo-location is approximate. Then, based on the initial geo-location, it is possible to calculate the LOS adjustment and re-image the target. Due to errors in GPS/INS and elevation of the target, there will be a re-projection error between the actual points of the target and the calculated projection ones during the reimaging. Third, an algorithm is proposed to deal with the reprojection error and obtain a new optimized target geo-location. Repeat the above process, and the target geo-location estimation will converge on the true value.

### 3.2. The Proposed Algorithm

In the proposed work pattern, the LOS should be always maintained as pointing to the target. The earth-fixed target geo-location is a state variable in the state equation that is constant. According to the position of the target in the sensor coordinate system, the target geo-location is computed by a nonlinear measurement equation as an initial state, which includes variances in the model. The equation can be expressed as

(4) $$\{\begin{array}{c} x_{k}=\Phi_{k-1}x_{k-1}+W_{k-1}\\ z_{k}=h(x_{k})+V_{k} \end{array},$$

where $x_{k}=[\varphi_{k},\lambda_{k},h_{k}]$ is the geographical position of the target *G*; and $\varphi_{k}$, $\lambda_{k}$, and $h_{k}$ are the latitude, longitude, and geodetic height of the target *G* at time k, respectively. $z_{k}=\left[\begin{array}{cc} m_{k} & n_{k} \end{array}\right]$ is obtained from each of the remote sensing images as the measurements. $W_{k-1}$ represents the process noise and $V_{k}$ represents the observation noises, which in the covariance matrix are $Q_{k-1}$ and $R_{k}$. $\Phi_{k-1}$ is the state transition matrix. For a stationary target, $\Phi_{k-1}$ and $Q_{k}$ are expressed as

(5) $$\Phi_{k-1}=\left[\begin{array}{ccc} 1 & 0 & 0\\ 0 & 1 & 0\\ 0 & 0 & 1 \end{array}\right],Q_{k-1}=\left[\begin{array}{ccc} 0 & 0 & 0\\ 0 & 0 & 0\\ 0 & 0 & 0 \end{array}\right],$$

The position of the target in an image is obtained by image registration. Using SIFT to obtain the image features in the current image and previous image, then matching the features and optimizing the match results using the RANSAC algorithm. The offsets of the target between two continuous images can be calculated by the match information. By controlling the LOS using the offsets of the target, the target position can always be in the image center and the target deviates from the image center within 2 pixels. The observation noises, whose covariance matrix is $R_{k}$, can be assumed as

(6) $$R_{k}=\left(\begin{array}{cc} 2 & 0\\ 0 & 2 \end{array}\right),$$

In Equation (4), $\Phi_{k-1}$ is the state transition matrix, and $h(x_{k})$ is the measurement transition matrix that can be used to compute the predicted measurement from the predicted state. The measurement transition matrix $h(x_{k})$ can be expressed

(7) $$h(x_{k})=\frac{1}{a}\times {\left[\begin{array}{cc} x_{G^{\prime }|k}^{S} & y_{G^{\prime }|k}^{S} \end{array}\right]}^{T},$$

where $G_{S|k}^{\prime }={\left[\begin{array}{ccc} x_{G^{\prime }|k}^{S} & y_{G^{\prime }|k}^{S} & z_{G^{\prime }|k}^{S} \end{array}\right]}^{T}=-f/z_{G|k}^{S}\times G_{S|k}$ represents the target estimation position in image. According to Equation (A1) in Appendix A, the coordinate of the target in the *S* coordinate system can be expressed as

(8) $$\begin{array}{l} \left[\begin{array}{c} G_{S|k}\\ 1 \end{array}\right]=\left[\begin{array}{c} x_{G|k}^{S}\\ y_{G|k}^{S}\\ z_{G|k}^{S}\\ 1 \end{array}\right]=C_{P}^{S}\times C_{NED}^{P}\times C_{ECEF}^{NED}\times \left[\begin{array}{c} \left(R_{N}+h_{k}\right)\cos\varphi_{k}\cos\lambda_{k}\\ \left(R_{N}+h_{k}\right)\cos\varphi_{k}\sin\lambda_{k}\\ (R_{N}(1-e^{2})+h_{k})\sin\varphi_{k}\\ 1 \end{array}\right] \end{array}$$

**Figure 6:**
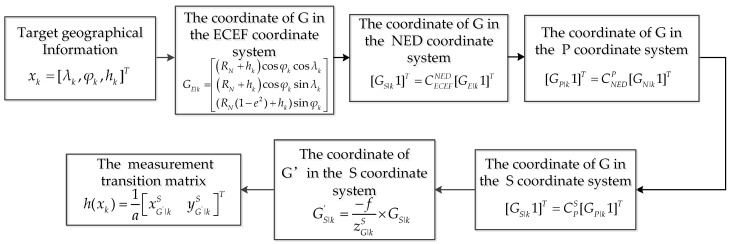
The solution process of the function $h(x_{k})$.

The solution process of the function $h(x_{k})$ is shown as Figure 6.

Since $h(x_{k})$ is a nonlinear matrix, the cubature Kalman filtering (CKF) method is adopted in our method [31]. CKF does not require the calculation of the Jacobian and Hessian matrices; the filter is easy to create, it quickly computes estimations with low complexity, and, in particular, it satisfies the system requirements for fast state estimation in real time. The steps of the weighted filtering algorithm are as follows:

Step 1: Determine the cubature sample point.

The cubature points and the corresponding weights are

(9) $$\begin{array}{l} \xi_{i}=\{\begin{array}{c} \sqrt{n}{\left[I\right]}^{\left(i\right)}\\ -\sqrt{n}{\left[I\right]}^{\left(i-n\right)} \end{array}\begin{array}{c} \mathrm{for}\;i=1,\cdots ,n\\ \;\mathrm{for}\;i=n+1,\cdots ,2n \end{array}\\ \omega_{i}=\frac{1}{2n},i=1,2,\cdots ,2n \end{array},$$

where i is the number of the cubature sample point. ${\left[I\right]}^{\left(i\right)}$ is the ith column of the n×n identity matrix.

Step 2: Information prediction.

We define $x_{0}$ as the initial state of the target geo-location, and $P_{0}$ is the initial matrix of the error variance matrix. $P_{k-1}$ is the error variance matrix at time k−1. By using the Cholesky decomposition approach [32], we get the equation

(10) $$P_{k-1}^{}=\sqrt{P_{k-1}}{\sqrt{P_{k-1}}}^{T},$$

$S_{k-1}$ is the square root matrix of $P_{k-1}$, So $S_{k-1}^{}=\sqrt{P_{k-1}}$.

Then, 2n cubature points can be obtained according to the following equation

(11) $$x_{i,k-1}^{}=\sqrt{P_{k-1}}\xi_{i}+x_{k-1},$$

We will use $S_{k-1}$ instead of $P_{k-1}$ at measurement updating step. Therefore, Equation (11) can be expressed

(12) $$x_{i,k-1}^{}=S_{k-1}\xi_{i}+x_{k-1}$$

The propagated cubature points can be expressed

(13) $$x_{i,k|k-1}^{}=\Phi_{k-1}x_{i,k-1}$$

Next, the one-step prediction can be completed according to Equation (13)

(14) $$x_{k|k-1}=\omega_{i}\sum_{i=1}^{2n}x_{i,k|k-1}^{}\;$$

(15) $$P_{k|k-1}=\omega_{i}\sum_{i=1}^{2n}(x_{i,k|k-1}^{}{\left(x_{i,k|k-1}^{}\right)}^{T}-x_{k|k-1}{\left(x_{k|k-1}\right)}^{T}\;)+Q_{k-1}$$

Step 3: QR decomposition.

In Equation (15), replacing $P_{k|k-1}$ with $S_{k|k-1}$ gives a new error variance matrix

(16) $$S_{k|k-1}^{}S_{k|k-1}^{T}=[\begin{array}{cc} x_{k|k-1}^{*} & S_{Q_{k-1}}^{} \end{array}]\left[\begin{array}{c} {\left(x_{k|k-1}^{*}\right)}^{T}\\ S_{Q_{k-1}}^{T} \end{array}\right]$$

(17) $$Q_{k-1}=S_{Q_{k-1}}S_{Q_{k-1}}^{T}$$

where $x_{k|k-1}^{*}=\frac{1}{\sqrt{2n}}[x_{1,k|k-1}^{}-x_{k|k-1}^{},\cdots ,x_{2n,k|k-1}^{}-x_{k|k-1}^{}]$ [33], $S_{Q_{k-1}}$ is the square root matrix of $Q_{k-1}$. $A_{2n\times n}$ is defined as $A_{2n\times n}={\left[\begin{array}{cc} {\left(x_{k|k-1}^{*}\right)}^{T} & S_{Q_{k-1}}^{T} \end{array}\right]}^{T}$, after QR decomposition, $A_{2n\times n}$ can be written as

(18) $$\{\begin{array}{c} A_{2n\times n}={\hat{Q}}_{2n\times n}{\hat{R}}_{n\times n}\\ I_{n\times n}={\hat{Q}}^{T}{}_{2n\times n}{\hat{Q}}_{2n\times n} \end{array},$$

where ${\hat{R}}_{n\times n}$ is the nonsingular upper or lower triangular matrix; thus, Equation (16) can be rewritten as

(19) $$\begin{array}{ll} S_{k|k-1}^{}S_{k|k-1}^{T} & =A_{2n\times n}^{T}A_{2n\times n}\\ & =({\hat{Q}}_{2n\times n}{\hat{R}}_{n\times n}{)}^{T}{\hat{Q}}_{2n\times n}{\hat{R}}_{n\times n}\\ & ={\hat{R}}^{T}{}_{n\times n}{\hat{R}}_{n\times n} \end{array},$$

where the one-step prediction of $S_{k|k-1}^{}$ can be expressed as $S_{k|k-1}^{}={\hat{R}}_{n\times n}^{T}$.

The QR decomposition can be derived from the orthogonalization of Gram–Schmidt, $S_{k-1}$ can be simplified as Equation (20) [34]

(20) $$S_{k|k-1}^{}=Tria(\left[x_{k|k-1}^{*},S_{Q_{k-1}}\right]),$$

In Equation (20), Tria(·) is the triangulation operation.

Step 4: Measurement updating.

After QR composition to transmit the square root factor of the covariance matrix, the 2n cubature points can be updated as

(21) $$x_{i,k|k-1}^{*}=x_{k|k-1}+S_{k|k-1}\xi_{i},$$

The updated cubature points can be transformed into the forms below based on the measurement function

(22) $$z_{i,k|k-1}^{}=h(x_{i,k|k-1}^{*}),$$

(23) $$z_{k|k-1}^{}=\omega_{i}\sum_{i=1}^{2n}z_{i,k|k-1}^{},$$

where i=1,2,⋯,2n, $z_{i,k|k-1}^{}$ are the transformed points. 

Then the square root of prediction covariance can be obtained by QR decomposition:

(24) $$S_{zz,k|k-1}^{}=Tria(\left[z_{k|k-1}^{*},S_{R_{k}}\right]),$$

where $z_{k|k-1}^{*}=\frac{1}{\sqrt{2n}}\left[z_{1,k|k-1}-z_{k|k-1},z_{1,k|k-1}-z_{k|k-1},\cdots ,z_{2n,k|k-1}-z_{k|k-1}\right]$, the measured random noise whose covariance matrix $R_{k}$ is defined as $R_{k}=S_{R_{k}}S_{R_{k}}^{T}$.

The cross variance matrix and the filter gain can be obtained according to Equations (25) and (26) below

(25) $$P_{xz,k|k-1}=\chi_{k|k-1}{\left(z_{k|k-1}^{*}\right)}^{T},$$

(26) $$K_{k}=(P_{xz,k|k-1}/S_{zz,k|k-1}^{T})/S_{zz,k|k-1}^{},$$

where $\chi_{k|k-1}=\frac{1}{\sqrt{2n}}\left[x_{1,k|k-1}^{*}-x_{k|k-1},x_{2,k|k-1}^{*}-x_{k|k-1},\cdots ,x_{2n,k|k-1}^{*}-x_{k|k-1}\right]$.

In the final step, the state estimation and the square root of error variance at time k can be updated as

(27) $$x_{k|k}=x_{k|k-1}+K_{k}(z_{k}-z_{k|k-1}),$$

(28) $$S_{k|k}=Tria(\left[\begin{array}{cc} \chi_{k|k-1}-K_{k}z_{k|k-1}^{*} & K_{k}S_{R_{k}} \end{array}\right]),$$

**Figure 7:**
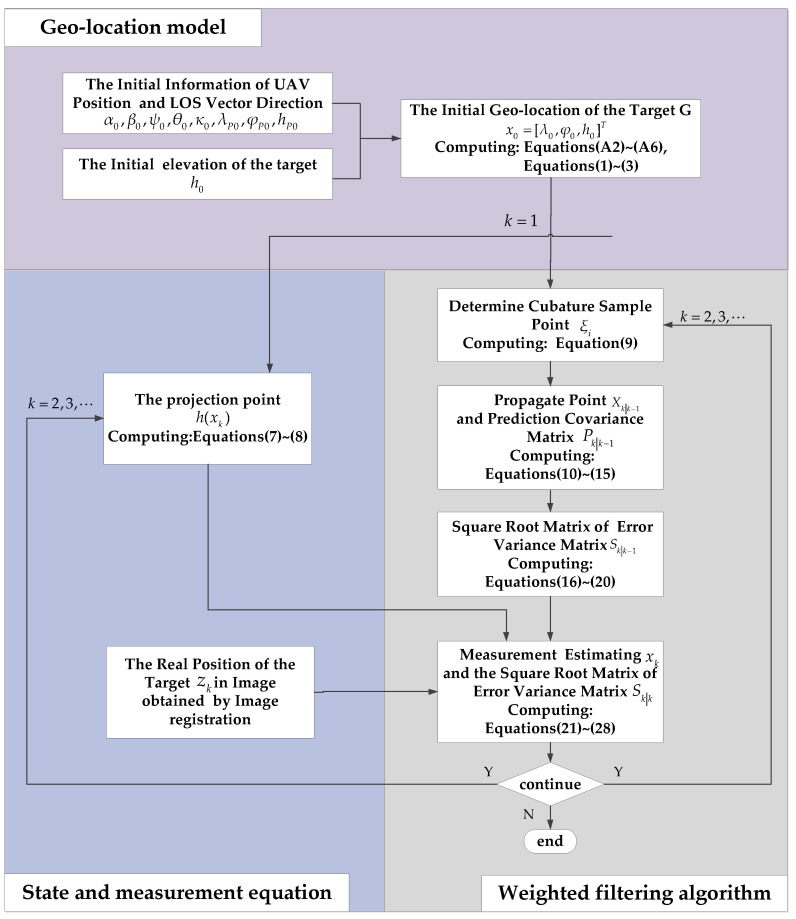
Flowchart of the proposed method.

A flowchart of the proposed method is shown in Figure 7. First, according to the information of GPS/INS and the LOS vector direction, the initial target geo-location $x_{0}$ can be calculated by the traditional method. Second, according to $x_{0}$, compute the projection point $h(x_{k})$ and reimage the target. The actual point $z_{k}$ can be obtained using image recognition. Third, by updating the state estimation, $x_{k+1}$ is obtained. Through the proposed iterative method, the geo-location of the target can be accurately estimated.

## 4. Experiments

The accuracy and robustness of the proposed method were verified by both simulation and flight experiment. First, the influencing factors on the proposed method were analyzed using the Monte-Carlo method. Moreover, the proposed method, the traditional method, DEM method, and the building target method were compared. Finally, the proposed method was applied in a real flight.

### 4.1. Simulation

The geo-location accuracy of the proposed method is affected by the flight height, the off-nadir looking angle, target elevation, UAV position, and LOS vector direction. The Monte-Carlo method was used to analyze the target geo-location error of the proposed method. The parameters of the simulation are summarized in Table 1. The geo-location error is defined as $1/N\sum_{k=1}^{N}\varepsilon_{k}$ in 1000 times simulation, where $\varepsilon_{k}$ is the geo-location error at each time. Five simulated experiments were performed.

#### 4.1.1. Effect of Flight Heights and Off-Nadir Looking Angle on Geo-Location Accuracy

**Figure 8:**
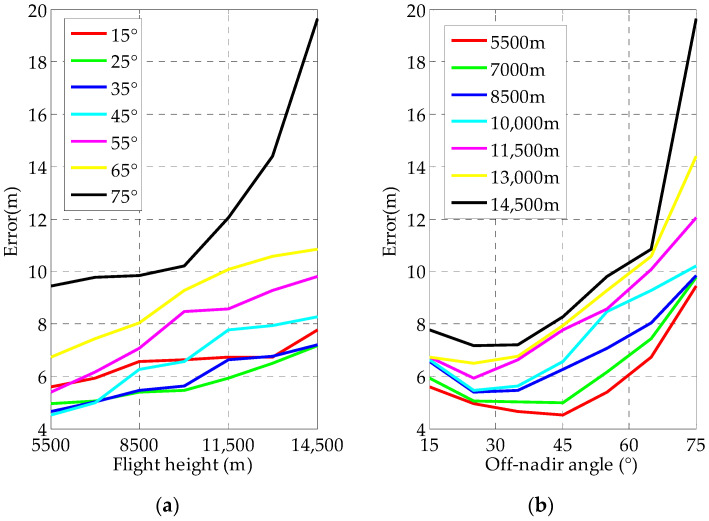
Flowchart of the proposed method. Influence of flight heights and off-nadir looking angle on geo-location: (**a**) Geo-location error curves with different flight heights when the off-nadir looking angle was changed from 15° to 75°; (**b**) Geo-location error curves with different off-nadir looking angles when the flight height was changed from 5500 m to 14,500 m.

The geo-location accuracy for different flight heights and off-nadir looking angles are shown in Figure 8.

Judging from the simulation results, three conclusions about the influence of the flight height and the off-nadir looking angle on the geo-location accuracy are as follows:

1. The geo-location accuracy is decreased with increasing flight height.
2. The influence of the off-nadir looking angle on the geo-location accuracy is increased with the increment of the flight height.
3. Even at a flight height of 14,500 m and an off-nadir looking angle of 75°, the target geo-location error is less than 20 m.

#### 4.1.2. Effect of Target Elevation on Geo-Location Accuracy

**Figure 9:**
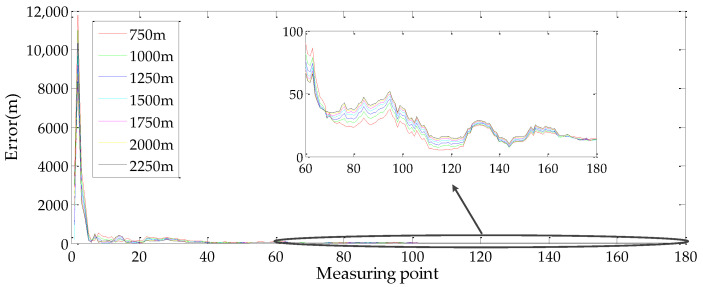
Influence of the target elevation error on the geo-location accuracy.

When the initial value of the target elevation changes from 750 m to 2250 m, the target elevation error correspondingly changes from −801 m to 699 m, the simulation results are shown in Figure 9. It can be seen that the target elevation error has little influence on the proposed method.

#### 4.1.3. Effect of UAV Position and LOS Vector Direction on Geo-Location Accuracy

**Figure 10:**
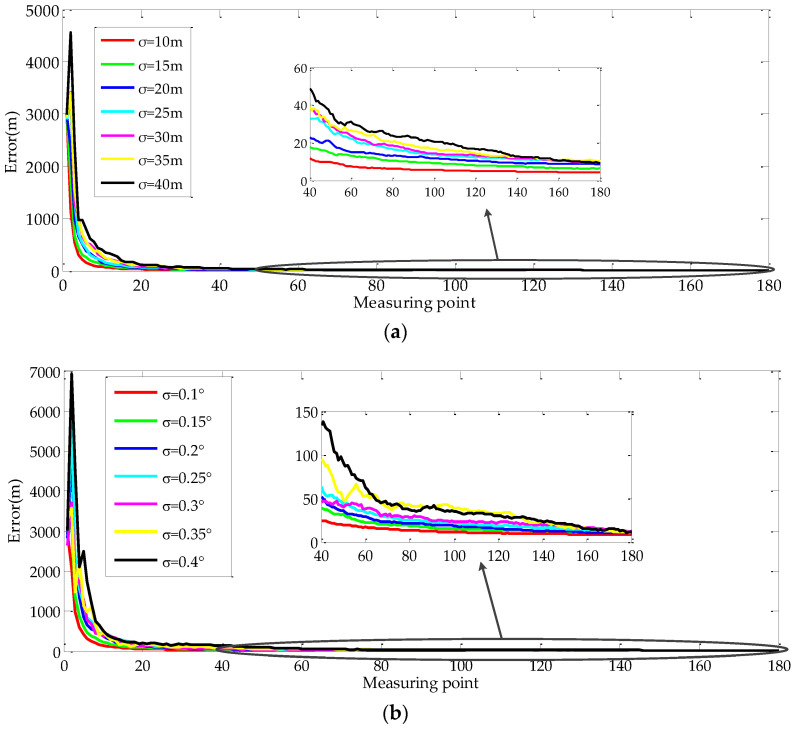
Influence of measurement variances on geo-location: (**a**) Geo-location error curves with different UAV position errors; (**b**) Geo-location error curves with different LOS vector direction errors.

When the UAV flies at a geodetic height of 10,000 m and the off-nadir looking angle is 75°, the influence of the UAV position and LOS vector direction are shown in Figure 10. It can be seen that the geo-location accuracy is mainly affected by the UAV position error and LOS vector direction error. The convergence speed and the geo-location accuracy decreased with the increase of the UAV position error and LOS vector direction error. After 180 times, the target geo-location error was less than 20 m.

#### 4.1.4. Comprehensive Simulation

In the simulation, it was assumed that the target *G* is located at the position (43.300000° N, 84.200000° E, 1551.00 m). The UAV flew around the target at a geodetic height of 10,000 m and the off-nadir looking angle was 75°.

The elevation error was less than 1500 m in rough terrain area where the target elevation was unknown. Therefore, $P_{0}$ can be assumed as

(29) $$P_{0}={\left[diag\left(0.015,0.015,1500\right)\right]}^{2},$$

The initial geo-location of the target was (43.303653° N, 84.195190° E, 1000.00 m), which was obtained by Equation (A2) and Equation (A3) in Appendix A. According to the ellipsoidal earth model, the geo-location error can be expressed as

(30) $$\varepsilon_{t}=\sqrt{{\left[\varepsilon_{\lambda }(R_{N}+h_{T})\cos\varphi_{T}\right]}^{2}+{\left[\varepsilon_{\varphi }(R_{M}+h_{T})\right]}^{2}+\varepsilon_{h}{}^{2}},$$

where $R_{M}=R_{E}(1-e^{2})/{\left(1-e^{2}\sin^{2}\varphi_{T}\right)}^{3/2}$ is the radius of the curvature in the principal vertical; and $\varepsilon_{\lambda }$, $\varepsilon_{\varphi }$, and $\varepsilon_{h}$ are the errors of the longitude, latitude, and geodetic height.

**Figure 11:**
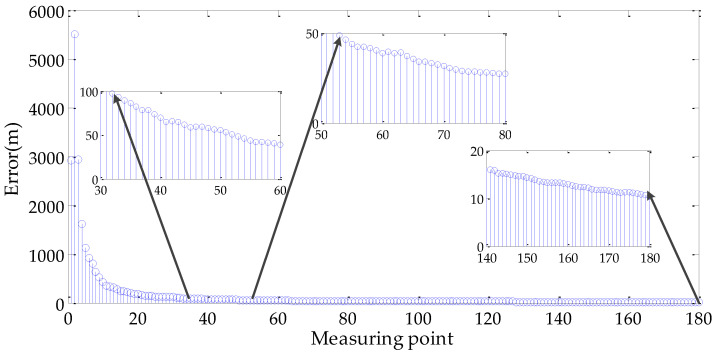
Geo-location error curves of the simulation.

The geo-location error curves are shown in Figure 11, when N is 1000. The geo-location error was less than 100 m after 32 measurements and reached within 50 m after 53 measurements. The final geo-location error was 10.44 m at 180 measurements, and the result of the target geo-location was (43.299979° N, 84.199981° E, 1549.52 m). 

#### 4.1.5. Comparison of Simulation Experiment with the Traditional Method

The geo-location error results were compared with the traditional method at a typical fight height of 10,000 m. When the target elevation error was 50 m, the corresponding results with the same measurement variances were as listed in Table 2.

According to Table 2, the method proposed in this paper significantly improved the geo-location accuracy of the target for LRORS.

#### 4.1.6. Comparison of the Simulation Experiment with the DEM Method

The geo-location error results were compared with DEM method [20]. The parameters used in the simulation process are presented in Table 3 (Table 1 in [20]).

**Figure 12:**
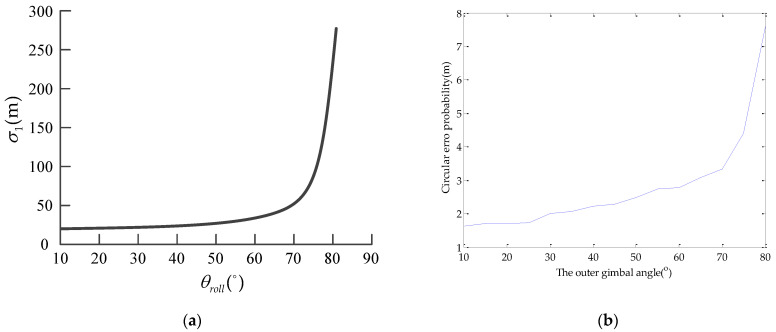
Circular error probability (CEP) of geo-location with different outer gimbal angle: (**a**) DEM method in [20], $\theta_{roll}$ is the outer gimbal angles and $\sigma_{1}$ is circular error probability; (**b**) the proposed method.

According to [20], when the outer gimbal angle was from 10° to 80°, the corresponding results with the same measurement variances were as shown in Figure 12.

It can be seen from Figure 12 that when the outer gimbal angle is 80°, the geo-location accuracy of the target calculated by DEM method was 180 m, while the geo-location accuracy obtained by our method was only 8 m. Compared with the DEM method, the accuracy of this method is improved by 22.5 times.

#### 4.1.7. Comparison of the Simulation Experiment with the Building Target Geo-Location Method

The geo-location error results were compared with the building target geo-location methods. The parameters used in the simulation process are presented in Table 4 (Table 1 in [22]).

**Figure 13:**
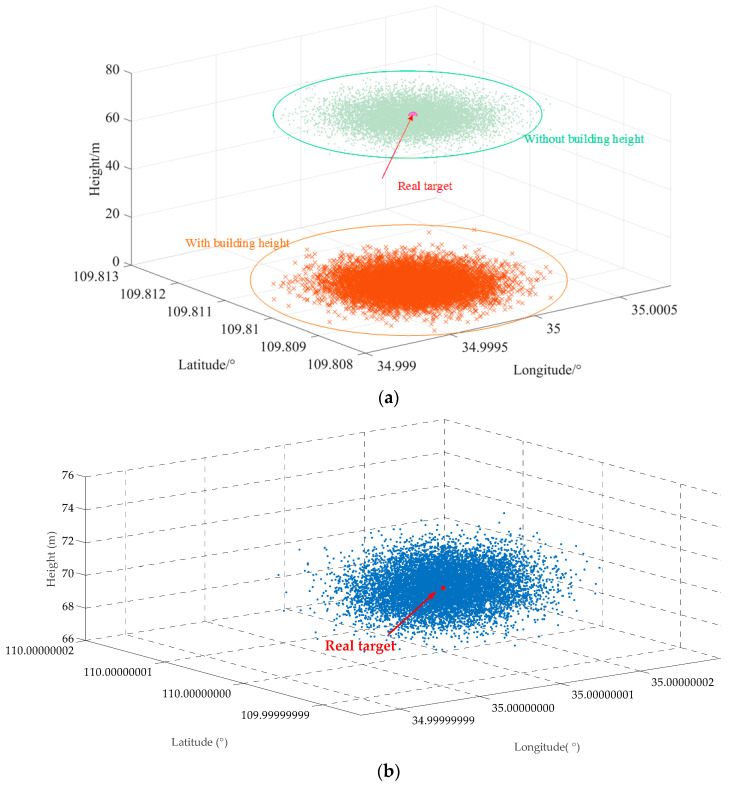
Schematic diagram of the distribution of geo-location results: (**a**) the building target geo-location method in [22]; (**b**) the proposed method.

According to [22], when the building target had a height of 70 m and the outer gimbal angle was 60°, the distribution of the geo-location results in 10,000 simulation experiments were as shown in Figure 13 and Table 5.

### 4.2. Flight Experiment and Results

**Figure 14:**
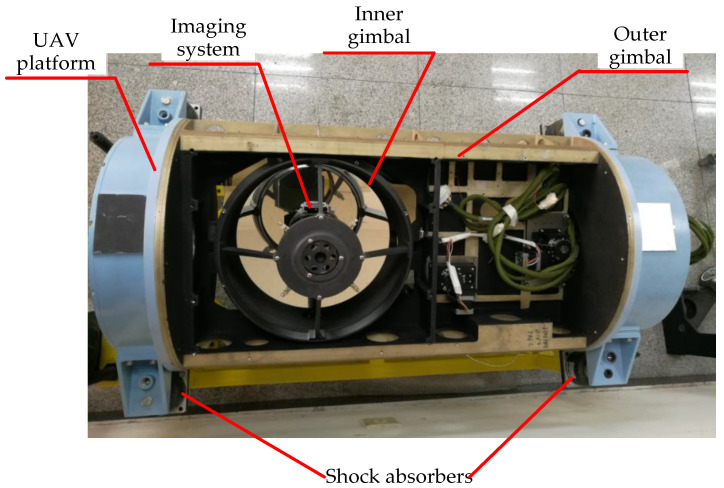
Photograph of the LRORS.

As shown in Figure 14, in order to validate the effectiveness of the proposed method, a flight experiment was carried out on the LRORS; the shock absorbers were used to connect with the UAV platform.

**Figure 15:**
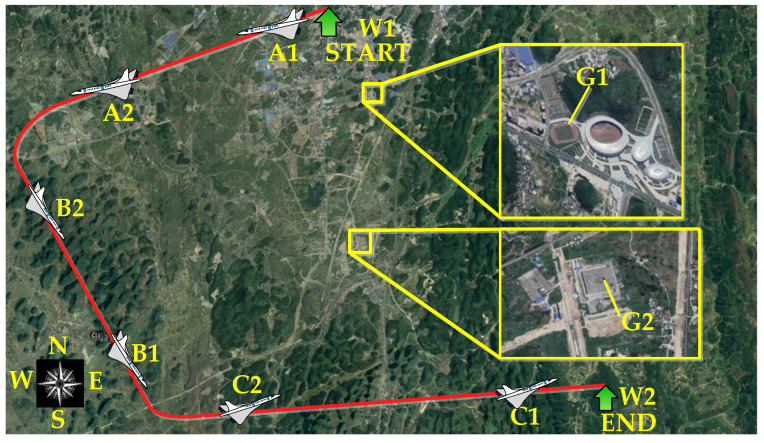
The flight path in Google Earth.

**Figure 16:**
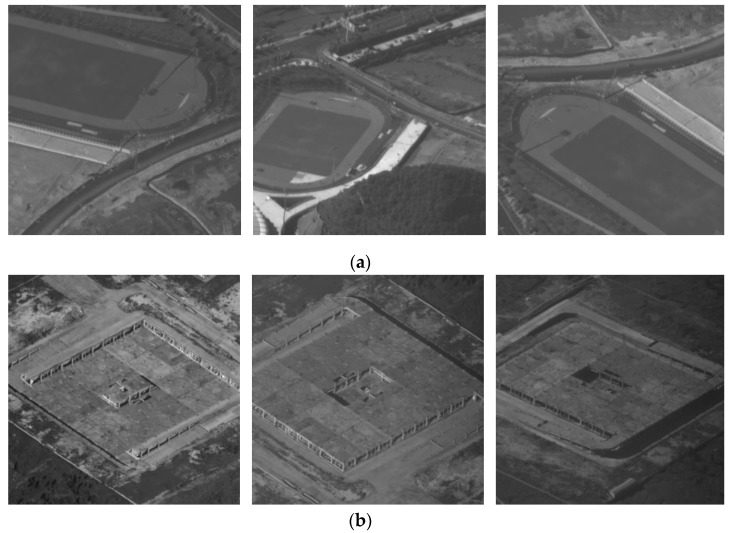
The remote sensing images obtained by the LRORS: (**a**) the remote sensing images of the target point G1; (**b**) the remote sensing images of the target point G2.

In Figure 15, W1 and W2 are the start point and the end point of the flight path. The targets G1 and G2 are measured by the LRORS when the UAV flies in a straight line, which were observed 100 times during the entire flight path. Remote sensing images of the targets are shown in Figure 16. Figure 16a shows the remote sensing images of the target point G1 obtained by the UAV in positions A1, B1, and C1, respectively. Figure 16b shows the remote sensing images of the target point G2 obtained by the UAV in positions A2, B2, and C2, respectively.

**Figure 17:**
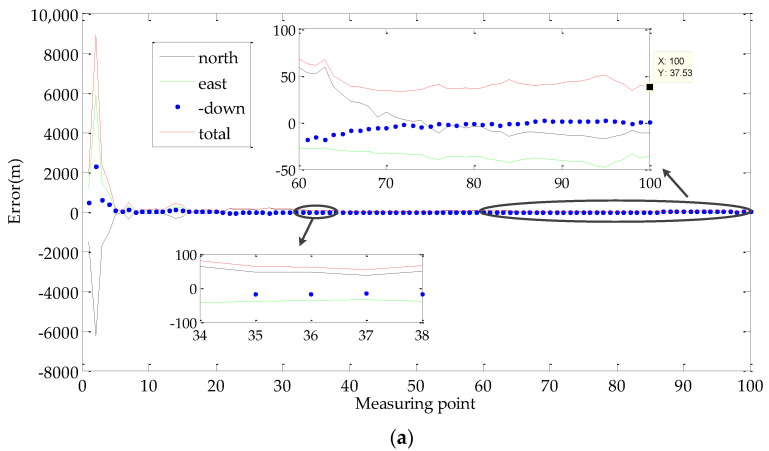

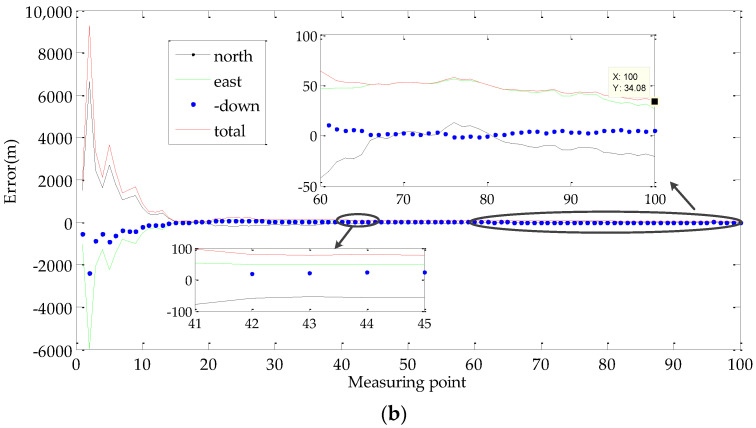
Results of geo-location in the flight test: (**a**) Target point G1; (**b**) Target point G2.

In order to verify the effectiveness of the proposed method, targets G1 and G2 were measured by the global navigation satellite system (GNSS) real time kinematic (RTK) method. The measuring equipment was survey-grade GNSS receivers I70 made by CHC- NAV. The positional accuracy for the points was less than 0.1 m and can be viewed as the standard value. The initial value of the target elevation was assumed equal to 800 m. The geo-location accuracy of the proposed method and the traditional method are shown in Figure 17 and Table 6.

In a comparison of two methods, it can be seen that the proposed method significantly improved the geo-location accuracy. We can see that the proposed method reduced the geo-location error from 1459.3 m to 37.53 m, which is improved by 38.8 times. It seems that our method could measure the elevation of the targets. As shown in Table 3, the elevation errors of the target points G1 and G2 were 2.65 m and 2.89 m, respectively.

## 5. Discussions

The traditional geo-location method heavily relies on the measurement precision of GPS/INS and target elevation accuracy, which restricts the target geo-location accuracy for LRORS. In order to improve the accuracy of target geo-location, the laser range finder method, DEM method, and image geo-registration method have been proposed in recent years, but they are inappropriate for long-range real-time target geo-location. The multiple-UAV method is difficult to implement in practical engineering. Focusing on the above mentioned problems, a set of work patterns and a novel geo-location method is proposed in this paper. There is an iterative process in the method, and the geo-location accuracy is improved greatly by repeatedly imaging the same stationary target point. The proposed method does not rely on the accuracy of GPS/INS and target elevation, is not limited by laser ranging distance, and does not need geographic reference data or the cooperative localization of multiple UAVs. The flight experimental data shows that our method can improve geo-location precision by 38.8 times compared with traditional methods.

The proposed method improved the geo-location accuracy through multiple observations on the same target. Theoretically, the method is not only applicable to long-distance targets, but also can improve the geo-location accuracy of targets at short distances. However, when observing the near-distance targets at different positions for multiple observations, the motion range of the LRORS gimbal will be significantly larger than observing a long-distance target, which requires a two-axis gimbal LRORS, to have a larger motion range.

As is well known, high-precision, real-time, and long-range target geo-location of UAVs cannot be separated from the support of positioning, navigation, and timing (PNT) technology. PNT technology has achieved rapid development in recent years. Therefore, it is necessary to discuss the influence of PNT on the geo-location accuracy of LRORS. On the one hand, the development of PNT will improve the geo-location accuracy of the traditional method. However, restricted by the target elevation, PNT technology has a limited improvement on the traditional method. On the other hand, the proposed method can realize geo-location without the elevation of the targets. PNT technology will improve the proposed method in two ways: first, reduce the observation times of the target; thus, reducing the motion range of a two-axis gimbal. Second, achieve a faster convergence and better accuracy.

## 6. Conclusions

A novel work pattern and algorithm was proposed in this paper. The geo-location accuracy is improved greatly by repeatedly imaging the same stationary target point. The proposed method can achieve high precision geo-location, without high-precision GPS/INS, multiple UAVs, and geographic reference data, such as standard maps, DEM, and so on. After verification using a Monte-Carlo simulation and flight experimental data, we can conclude that the proposed method has a better capability to improve the accuracy of target geo-location compared with the present methods. The results show that the proposed method can improve the geo-location accuracy by 38.8 times and 22.5 times comparing with traditional method and DEM method, respectively. The analysis results show that the proposed method has a strong timeliness and more extensive application value in practical engineering.

## Figures and Tables

**Table 1:** Measurement Variances in Geo-location.

	Error Type	Error Value
UAV position	latitude (north)	0.00018°
	longitude (east)	0.00024°
	geodetic height (down)	40 m
UAV attitude	yaw	0.3°
	pitch	0.1°
	roll	0.1°
gimbal angle	outer	0.01°
	inner	0.01°

**Table 2:** Geo-location error results of the proposed method and the traditional method.

Method	Off-Nadir Looking Angle	The Geo-Location Error			
		UAV Position Error	LOS Vector Direction Error	Target Elevation Error	Total Error
The traditional method	60°	91 m	24 m	99 m	151 m
	65°	111 m	30 m	117 m	182 m
	70°	141 m	46 m	147 m	237 m
	75°	191 m	69 m	195 m	316 m
The proposed method	60°	2.36 m	7.35 m	0.73 m	8.83 m
	65°	2.45 m	7.93 m	0.76 m	9.27 m
	70°	3.25 m	8.26 m	0.81 m	9.98 m
	75°	4.3554 m	9.52 m	0.87 m	10.44 m

**Table 3:** Simulation experiment parameters in [20].

	Error Type	Error Value
UAV position	latitude (north)	0.0001°
	longitude (east)	0.0001°
	geodetic height (down)	5 m
UAV attitude	yaw	0.02°
	pitch	0.01°
	roll	0.01°
gimbal angle	outer	0.006°
	inner	0.006°
UAV flight	height	18,000 m

**Table 4:** Simulation experiment parameters in [22].

	Error Type	Error Value
UAV position	latitude (north)	0.0001°
	longitude (east)	0.0001°
	geodetic height (down)	10 m
UAV attitude	yaw	0.06°
	pitch	0.02°
	roll	0.02°
gimbal angle	outer	0.006°
	inner	0.006°
UAV flight	height	10,000 m

**Table 5:** Geo-location error results.

Method	The Average Position Error of the Latitude	The Average Position Error of the Longitude
The building target geo-location method [22]	2.8738 × 10^−6^°	2.3203 × 10^−6^°
The proposed method	3.6398 × 10^−9^°	4.3882 × 10^−9^°

**Table 6:** Geo-location error results in the flight test.

Method	Error Type	Target Point G1	Target Point G2
Geographical position standard value by GNSS	latitude (north)	26.221386°	26.184767°
	longitude (east)	105.894206°	105.857411°
	geodetic height (down)	1367.89 m	1324.67 m
The proposed method	latitude (north)	26.221087°	26.184489°
	longitude (east)	105.894025°	105.857283°
	geodetic height (down)	1365.24 m	1321.78 m
	total error	37.53 m	34.08 m
The traditional method	latitude (north)	26.2135937°	26.1955568°
	longitude (east)	105.8838978°	105.8639344°
	geodetic height (down)	800 m	800 m
	total error	1459.3 m	1459.5 m

**Figure A1:**
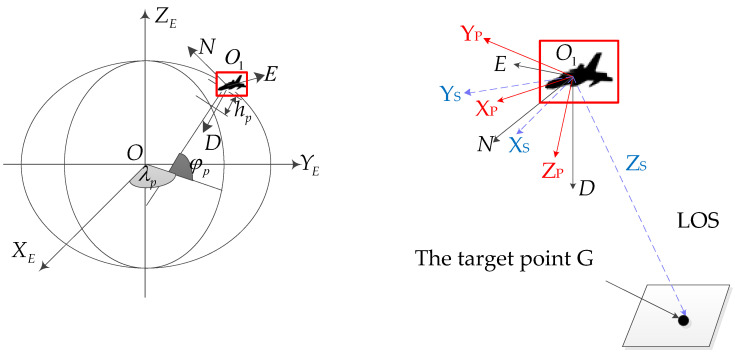
Schematic diagram of the coordinate systems.

**Figure A2:**
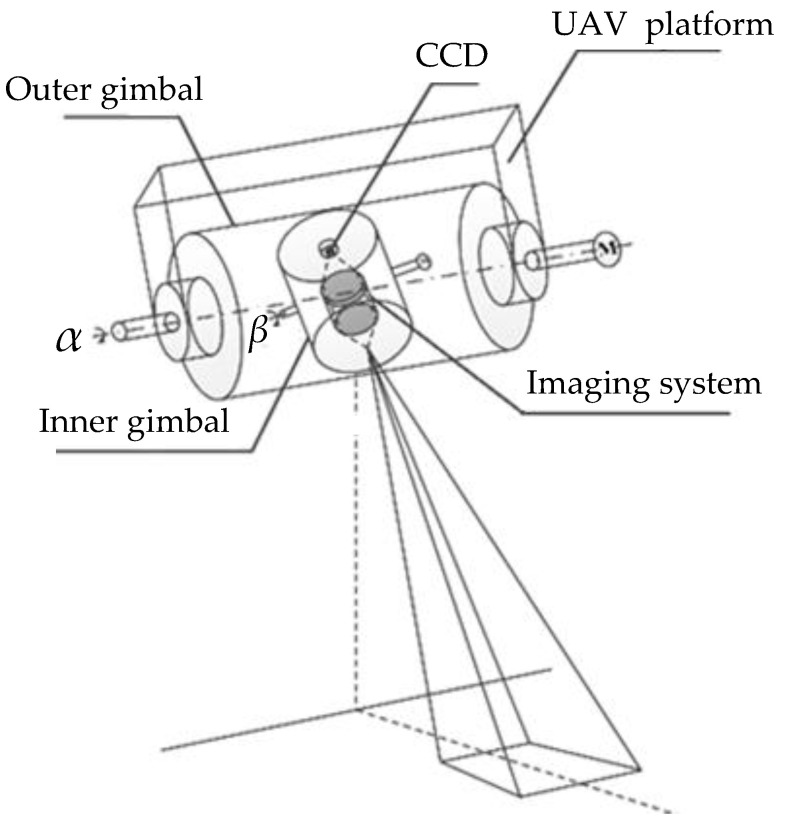
The structure of the LRORS.

## Appendix A

The coordinates systems involved in this section are shown in Figure A1. *O*-*X_E_Y_E_Z_E_* is the ECEF coordinate system, which is defined in WGS-84 and has its origin at the earth’s geometric center. *O*_1_-NED is the NED coordinate system, whose origin *O*_1_ is the center of UAV platform. *O*_1_-*X_P_Y_P_Z_P_* and *O*_1_-*X_S_Y_S_Z_S_* are the *P* coordinate system and *S* coordinate system, respectively.

The geo-location of point *G* can be expressed as the longitude, latitude, and geodetic height (recorded *λ_G_*, *φ_G_*, and *h_G_*, respectively). The point *G* in the ECEF coordinate system can be expressed as Equation (A1) [16,35,36,37,38].

(A1) $$\left[\begin{array}{c} x_{G}^{E}\\ y_{G}^{E}\\ z_{G}^{E} \end{array}\right]=\left[\begin{array}{c} \left(R_{N}+h_{G}\right)\cos\varphi_{G}\cos\lambda_{G}\\ \left(R_{N}+h_{G}\right)\cos\varphi_{G}\sin\lambda_{G}\\ (R_{N}(1-e^{2})+h_{G})\sin\varphi_{G} \end{array}\right]$$

where $e=\sqrt{R_{E}{}^{2}-R_{P}{}^{2}}/R_{E}$ is the first eccentricity of WGS-84 coordinates reference ellipsoid, and $R_{E}=6,378,137\;m$ and $R_{P}=6,356,752.3142\;m$ are the long and short half axles of ellipsoid, respectively. $R_{N}=R_{E}/\sqrt{1-e^{2}\sin^{2}\varphi }$ is the radius of the curvature in prime vertical.

According to the ellipsoidal earth model, the latitude of the northern hemisphere is positive and the latitude of the southern is negative. The latitude and geodetic height can be solved by the following iteration equation:

(A2) $$\begin{array}{l} \left\{ \begin{array}{l} R_{N}{}_{0}=R_{E}\\ h_{0}=[(x^{E}{)}^{2}+(y^{E}{)}^{2}+(z^{E}{)}^{2}{]}^{\frac{1}{2}}-(R_{E}R_{p}{)}^{\frac{1}{2}}\\ \varphi_{0}=\tan^{-1}\left[\frac{z^{E}}{\sqrt{(x^{E}{)}^{2}+(y^{E}{)}^{2}}}{\left(1-\frac{e^{2}N_{0}}{\left(N_{0}+h_{0}\right)}\right)}^{-1}\right] \end{array}\right.\\ \left\{ \begin{array}{l} R_{Ni}=R_{E}(1-e^{2}\sin^{2}\varphi_{i-1}{)}^{-\frac{1}{2}}\\ h_{i}=\frac{\sqrt{{\left(x^{E}\right)}^{2}+{\left(y^{E}\right)}^{2}}}{\cos\varphi_{i-1}}-R_{Ni}\\ \varphi_{i}=\tan^{-1}\left[\frac{z^{E}}{\sqrt{{\left(x^{E}\right)}^{2}+{\left(y^{E}\right)}^{2}}}{\left(1-\frac{e^{2}R_{Ni}}{\left(R_{Ni}+h_{i}\right)}\right)}^{-1}\right] \end{array}\right. \end{array},$$

Generally, when the iterations are over four, the computing accuracy of the latitude and geodetic height are higher than 0.00001 and 0.001 m, respectively [20].

According to the ellipsoidal earth model, the longitude of the eastern hemisphere is positive and the longitude of the western hemisphere is negative. The longitude can be solved using the following equation [20]:

(A3) $$\lambda =\{\begin{array}{c} \lambda_{0}\\ \lambda_{0}+\pi \\ \lambda_{0}-\pi \end{array}\;\begin{array}{c} x^{E}>0\\ x^{E}<0,\lambda_{0}<0\\ x^{E}<0,\lambda_{0}>0 \end{array}$$

where $\lambda_{0}=\tan^{-1}(y^{E}/x^{E})$.

The matrix transforms from the ECEF coordinate system to NED coordinate system can be expressed as [16]

(A4) $$C_{ECEF}^{NED}=\left[\begin{array}{cccc} -S\varphi_{P}C\lambda_{P} & -S\varphi_{P}S\lambda_{P} & C\varphi_{P} & R_{NP}e^{2}S\varphi_{P}C\varphi_{P}\\ -S\lambda_{P} & C\lambda_{P} & 0 & 0\\ -C\varphi_{P}C\lambda_{P} & -C\varphi_{P}S\lambda_{P} & -S\varphi_{P} & R_{NP}+h_{P}-R_{NP}e^{2}S^{2}\varphi_{P}\\ 0 & 0 & 0 & 1 \end{array}\right],$$

where “sin” and “cos” are abbreviated to “*S*” and “*C*”, respectively, and *R_NP_* denotes the prime vertical radius of the curvature of the UAV platform, the geographical position *λ_P_*, *φ_P_*, and *h_P_* are the longitude, latitude, and geodetic height of the UAV platform, respectively.

The matrix transformation from the NED coordinate system to the *P* coordinate system can be expressed as [17]

(A5) $$C_{NED}^{P}=\left[\begin{array}{cccc} C\theta C\psi & C\theta S\psi & -S\theta & 0\\ S\kappa S\theta C\psi -C\kappa S\psi & S\kappa S\theta S\psi +C\kappa C\psi & S\kappa C\theta & 0\\ C\kappa S\theta C\psi +S\kappa S\psi & C\kappa S\theta S\psi -S\kappa C\psi & C\kappa C\theta & 0\\ 0 & 0 & 0 & 1 \end{array}\right],$$

where the attitude angles ψ, θ, and κ are yaw angles, pitch angles, and roll angles of the UAV platform, respectively.

Generally, a LRORS consists of an imaging system and a two-axis gimbal. The imaging system is installed in a two axis gimbal which is connected with the UAV platform, and the basic structure is shown in Figure A2.

The *S* coordinate system has its origin at the principal point of the imaging system, and the Z_S_ axis is the LOS of the imaging system. When the outer and inner gimbal angles are α and β, the matrix transformation from the *P* coordinate system to *S* coordinate system can be expressed as [20]

(A6) $$C_{P}^{S}=\left[\begin{array}{cccc} C\beta & S\beta S\alpha & -S\beta C\alpha & 0\\ 0 & C\alpha & S\alpha & 0\\ S\beta & -C\beta S\alpha & C\beta C\alpha & 0\\ 0 & 0 & 0 & 1 \end{array}\right]$$
